# Interaction of TNF with Angiotensin II Contributes to Mitochondrial Oxidative Stress and Cardiac Damage in Rats

**DOI:** 10.1371/journal.pone.0046568

**Published:** 2012-10-09

**Authors:** Nithya Mariappan, Carrie M. Elks, Masudul Haque, Joseph Francis

**Affiliations:** 1 Department of Comparative Biomedical Sciences, Louisiana State University School of Veterinary Medicine, Baton Rouge, Louisiana, United States of America; 2 Nutritional Neuroscience and Aging Laboratory, Pennington Biomedical Research Center, Louisiana State University System, Baton Rouge, Louisiana, United States of America; National Institutes of Health, United States of America

## Abstract

Recent evidence suggests that tumor necrosis factor alpha (TNF) and angiotensin II (ANGII) induce oxidative stress contribute to cardiovascular disease progression. Here, we examined whether an interaction between TNF and ANGII contributes to altered cardiac mitochondrial biogenesis and ATP production to cause cardiac damage in rats. Rats received intraperitoneal injections of TNF (30 µg/kg), TNF + losartan (LOS, 1 mg/kg), or vehicle for 5 days. Left ventricular (LV) function was measured using echocardiography. Rats were sacrificed and LV tissues removed for gene expression, electron paramagnetic resonance and mitochondrial assays. TNF administration significantly increased expression of the NADPH oxidase subunit, gp91phox, and the angiotensin type 1 receptor (AT-1R) and decreased eNOS in the LV of rats. Rats that received TNF only had increased production rates of superoxide, peroxynitrite and total reactive oxygen species (ROS) in the cytosol and increased production rates of superoxide and hydrogen peroxide in mitochondria. Decreased activities of mitochondrial complexes I, II, and III and mitochondrial genes were observed in rats given TNF. In addition, TNF administration also resulted in a decrease in fractional shortening and an increase in Tei index, suggesting diastolic dysfunction. TNF administration with concomitant LOS treatment attenuated mitochondrial damage, restored cardiac function, and decreased expression of AT1-R and NADPH oxidase subunits. Mitochondrial biogenesis and function is severely impaired by TNF as evidenced by downregulation of mitochondrial genes and increased free radical production, and may contribute to cardiac damage. These defects are independent of the downregulation of mitochondrial gene expression, suggesting novel mechanisms for mitochondrial dysfunction in rats given TNF.

## Introduction

The renin-angiotensin system (RAS) plays an important role in the pathophysiology of cardiovascular disease. Over-activation of the local tissue RAS is one of the most common pathophysiological mechanisms contributing to the progression of cardiovascular diseases, and is also an important target for current pharmaceutical therapies. Approximately 50 million Americans suffer from hypertension; several etiological agents, including obesity and lifestyle changes, have been attributed to the progression of this disease and its effects on target organs. Hence, one of the main objectives in treating hypertension is end organ protection. Several Clinical trials using calcium channel blockers and RAS antagonists have been effective [1], [2]; however, the clinical course of hypertension is progressive, and the need for new and innovative approaches for its treatment remains critical.

A growing body of evidence suggests that the main effector peptide of the RAS, angiotensin II (ANGII), induces inflammatory molecules and contributes to the pathophysiology of cardiovascular disease [3]–[5]. More importantly, proinflammatory cytokines (PIC), such as tumor necrosis factor alpha (TNF), interleukin (IL)-1β and IL-6, play important roles in hypertension and heart diseases [6]–[8]. Although several PIC are upregulated in hypertension, we have focused on TNF, since it is generally the first cytokine that is upregulated in disease and since it also induces the production of several other cytokines [9], [10]. Recent findings from our lab suggest that high concentrations of TNF induce cardiac damage [11], [12]. It is well-established that the RAS plays an important role in blood pressure regulation and also in the pathophysiologies of heart and kidney disease [7], [13]. Both PIC and RAS have been shown to induce and respond to oxidative stress. Furthermore, data from our lab suggest that mice lacking the gene for TNF, when treated with ANGII, demonstrate attenuated hypertensive and hypertrophic responses [7]. Although studies in experimental animal models indicate that both TNF and ANGII are involved in the pathophysiology of hypertension [14], [15], possible interactions between these factors have not been explored at the mitochondrial level.

Cardiac function is highly dependent on mitochondrial ATP supply; hence, the heart is especially susceptible to the disruption of mitochondrial energy production. Mitochondrial reactive oxygen species (ROS) are involved in cell signaling, but are also potential mediators of oxidative stress. Oxidative stress mediated by activation of the ANGII type-1 receptor (AT-1R) plays a crucial role in the progression of cardiovascular diseases [16]–[19]. Dysfunctional mitochondria, in turn, produce excessive amounts of ROS such as superoxide (O_2_^•−^), hydrogen peroxide (H_2_O_2_), and peroxynitrite (OONO^−^). This overproduction of mitochondrial ROS contributes to cardiac dysfunction. In addition to ROS formation, reduced expression of mitochondrial genes and proteins can contribute to mitochondrial dysfunction.

The objective of the study was to determine whether increased ROS production, induced by TNF and ANGII, which is associated with ATP deficit, could directly cause mitochondrial oxidative damage, leading to depletion/reduction of mitochondrial genes and proteins, dysfunction of the mitochondrial electron transport system, and consequently, cardiac damage. Results from this study will help us elucidate the role played by TNF in inducing oxidative stress by examining the superoxide producing machinery in the heart, and its contribution to cardiac dysfunction. In this context, the purpose of this study was to investigate whether an AT-1R blocker (losartan) could attenuate the functional and structural changes that occur in cardiac mitochondria upon TNF induction. Identifying the links between TNF, ANGII, and oxidative stress at the mitochondrial level in contributing to cardiac damage may lead to a better understanding of the progression of cardiovascular disease and, ultimately, lead to new and effective treatment strategies.

## Materials and Methods

### Ethics Statement

All experimental procedures were in compliance with all applicable principles set forth in the National Institutes of Health Guide for the Care and Use of Laboratory Animals (Publication No. 85-23, revised 1996). This study was approved by the Institutional Animal Care and Use Committee of the Louisiana State University School of Veterinary Medicine (protocol approval number 09-008).

### Experimental Protocol

Studies were conducted using adult male Sprague–Dawley rats (325–350 g; n = 8 per group) obtained from Harlan (Indianapolis, IN, USA). Animals were housed in temperature-(23±2°C) and light-controlled (12 h light/dark cycle) animal quarters; standard rat chow and water were provided *ad libitum*. Rats were divided into 3 groups. One group of rats received TNF at a dose of 30 µg/kg, ip, the second group received normal saline vehicle, and group 3 received TNF + losartan (LOS, 1 mg/kg, ip), for 5 days. Rats were sacrificed by carbon dioxide inhalation, and left ventricle (LV) samples were collected for gene expression and measurement of oxidative stress markers. Mitochondria were isolated by differential centrifugation for functional studies. Electron paramagnetic resonance (EPR) spectroscopy was used to measure free radical production in the cytosolic and mitochondrial fractions. The structural integrity of mitochondrial membranes was measured using swelling assay and transmission electron microscopy (TEM) analysis.

### Blood Pressure

Blood pressure were measured noninvasively using a Coda 6 Blood Pressure System (Kent Scientific, Torrington, CT), which utilizes a tail-cuff occlusion method and volume pressure recording (VPR) sensor technology. In this system, unanesthtized rats from each group were warmed to an ambient temperature of 30°C by placing them in a holding device mounted on a thermostatically controlled warming plate. Rats were allowed to habituate to this procedure for 3 days prior to each experiment. Blood pressure values were averaged from at least six consecutive cycles per day obtained from each rat.

### Echocardiography

Cardiac function was measured as described previously [11], [20], [21]. Rats were anesthetized with isoflurane to facilitate positioning for echocardiographic studies. LV end diastolic volume (LVEDV), end systolic volume (LVESV), intraventricular wall thickness and posterior wall thickness were measured in systole and diastole to determine the presence of hypertrophy.

### Electron Spin Resonance Studies

Total LV ROS and superoxide production rates were measured using EPR as described previously [12], [22]. Three different spin probes were used for EPR studies. 1-Hydroxy-3-methoxycarbonyl-2,2,5,5-tetramethyl-pyrrolidine (CMH) was used to measure tissue ROS and superoxide O_2_^•−^, 1-hydroxy-3-carboxy-2,2,5,5-tetramethylpyrrolidine (CPH) was used for measurement of tissue peroxynitrite (OONO^−^); and 1-hydroxy-4-phosphono-oxy-2,2,6,6-tetramethyl-piperidine (PPH) was used for mitochondrial O_2_^•−^ and hydrogen peroxide (H_2_O_2_) studies [23]. Briefly, pieces of LV tissues were incubated at 37°C with CMH (200 µM) for 30 min for ROS measurement; PEG-SOD (50 U/µl) for 30 min, then CMH (200 µM) for an additional 30 min for O_2_^•−^ measurement; or CPH (500 µM) for 30 min for OONO^−^ measurement. Aliquots of incubated probe media were then taken in 50-µl disposable glass capillary tubes (Noxygen Science Transfer and Diagnostics) for determination of LV ROS, O_2_^•−^, or OONO^−^ production. All EPR measurements were performed using an EMX ESR eScan BenchTop spectrometer and super-high quality factor microwave cavity (Bruker Company, Germany).

### RNA Isolation and Real-Time RT-PCR

Total RNA extraction, cDNA synthesis, and real-time RT-PCR were performed as previously described [11]. Rat primer sequences appear in Table 1.

**Table 1:** Rat primers used for RT-PCR.

Gene	TNF	Antisense
**GAPDH**	agacagccgcatcttcttgt	cttgccgtgggtagagtcat
**gp91phox**	cggaatcctctccttcct	gcattcacacaccactccac
**NOX4**	ttctacatgctgctgctgct	aaaaccctccaggcaaagat
**AT-1R**	caacctccagcaatcctttc	cccaaatccatacagccact
**TNF-α**	gtcgtagcaaaccaccaagc	tgtgggtgaggagcacatag
**eNOS**	ggcatacagaacccaggatg	ggatgcaaggcaagttagga
**iNOS**	ccttgttcagctacgccttc	ggtatgcccgagttctttca
**CPT1**	ctcagcctctacggcaaatc	tgcccatgagtgttctgtgt
**CPT2**	ctaatcccaaggtgcttcca	cttcagttgggctctt
**PGC1α**	aagcaggtctctccttgcag	ccatcccgtagttcactggt
**PGC1β**	tggatgagctttcactgctg	tggatgagctttcactgctg
**UCP3**	ggcccaacatcacaagaaac	agctccaaaggcagagacaa

### Isolation of Mitochondria and Mitochondrial Functional Studies

LV mitochondria were isolated by differential centrifugation of heart homogenates as described previously [11]; for assessment of permeability transition pore opening, mitochondrial swelling was measured as described previously [11], [22]. Ultrastructural examination of isolated mitochondrial preparations was performed as described before [22].

### Western Blotting

Protein expression in mitochondria was analyzed by western blotting as previously described [11], [22], using anti-ANT, anti- cytochrome c and anti-VDAC antibodies (Santa Cruz Biotechnology). The band intensities were quantified using a BioRad ChemiDoc imaging system and normalized to VDAC.

### Mitochondrial O2^•−^ and H_2_O_2_ Production

Mitochondrial O_2_^•−^ and H_2_O_2_ production in mitochondria were measured using EPR as described previously [12], [22]. [23]Aliquots of isolated LV mitochondria were probed with PPH (500 µM) alone or PPH and SOD (50 U/ml) for quantification of O_2_^•−^ production. Catalase (50 U/ml) was added to measure H_2_O_2_ formation. PPH allows the detection of extracellular and extra mitochondrial production of O_2_^•−^ [24]. PPH reacts with O_2_^•−^ to produce a stable PP^•^ nitroxide radical which can be detected with EPR [25]. After adequate mixing, 50 µl of mitochondria were taken in 50 µl glass capillary tubes. Mitochondrial O_2_^•−^ production and H_2_O_2_ production were determined by EPR under the same settings as were used for measurement of mitochondrial O_2_^•−^ and H_2_O_2_ production.

### Enzymatic and Respiratory Activities of Mitochondrial Complexes I–III

Measurements of mitochondrial complex I, II and III enzymatic activities were performed as previously described [12]. Briefly, aliquots of mitochondria were mixed with oxygenated KHB (20 mm Hg–pO_2_) containing 1 mM EGTA. Then, the oxygen spin label NOX-13.1-OS (5 µM), CMH (200 µM), and one of the following substrates were added to the mitochondrial suspension: 20 mM glutamate (complex I), 5 mM succinate (complex II), or 5 mM pyruvate (complex III). After adequate mixing, the samples were taken into capillary tube and mitochondrial complex I, II and III were measured using EPR. Activity of each mitochondrial complex was quantified by EPR under the same settings described previously [12], [22].

### Measurement of ATP and ADP/ATP Ratio

ATP production rates and ADP/ATP ratios were quantified in isolated mitochondria using a commercially available kit (BioVision, Mountain View, CA) as described previously [12], [22].

### Statistical Analyses

All data are expressed as mean ± SEM. Statistical analyses were performed using GraphPad Prism version 5.00 for Windows**.** Data were analyzed by ANOVA, followed by Bonferroni’s multiple comparison tests. In all cases, *p*<0.05 was considered statistically significant.

## Results

### Blood Pressure

Blood pressure measurements were obtained for all experimental animals during the 5 day treatment period. Administration of TNF with or without LOS did not have any effect on blood pressure parameters (Table 2).

**Table 2:** Blood pressure data from control and experimental groups.

Days	MAP mmHg		
	Control	TNF	TNF +LOS
1	111±1.57	110±0.55	105±0.23
2	108±0.69	115±0.11	110±0.05
3	109±0.33	110±0.22	105±0.05
4	112±0.88	110±0.02	110±0.11
5	114±0.66	115±0.22	114±0.23

Mean arterial pressures for control and experimental groups. No statistically significant differences were observed among mean arterial pressures during 5-day treatment period.

### Echocardiography

When compared to control animals, TNF treated rats had progressive increases in LV end-diastolic dimension (LVD), LV end-systolic dimension (LVS) and TEI index and decreases in fractional shortening (FS %) measurements (Table 3). Additionally, rats treated with TNF+LOS exhibited significant decreases in LVD, LVS and TIE index and increases in FS% when compared to rats given TNF only.

**Table 3:** Echocardiographic data from experimental groups.

Parameter	Control	TNF	TNF +LOS
IVSD (mm)	1.37±0.001	1.68±0.06	1.33±0.06
IVSS mm)	2.31±0.08	2.48±0.07	2.37±0.1
LVD (mm)	7.27±0.06	7.97±0.06*	7.34±0.1**$**
LVS (mm)	5.19±0.10	5.74±0.16*	5.27±0.1 **$**
PWD(mm)	1.29±0.02	1.42±0.04	1.34±0.1
PWS (mm)	2.18±0.026	2.24±0.09	2.27±0.04
FS%	36.86±0.72	26.85±0.45*	35.88±0.7**$**
HR	332.25±3.2	329.40±4.1	333.75±4.9
TEI	0.27±0.01	0.38±0.04*	0.29±0.003**$**

Echocardiographic analysis revealed that both left ventricular diastolic (LVD) and systolic (LVS) dimensions were significantly greater in TNF treated animals. TNF treatment also decreased fractional shortening (FS) and increased Tei index when compared to controls. Co treatment of TNF treated rats with losartan prevented these changes. IVSD, intraventricular septal thickness at end diastole; IVSS, intraventricular septal thickness at end systole; PWD, posterior wall thickness at end diastole; PWS, posterior wall thickness at end systole; HR, heart rate. Values are expressed as means ± SEM.

* p<0.05 vs. control;

$ p<0.05 vs.TN.

### EPR Measurements

**Figure 1:**
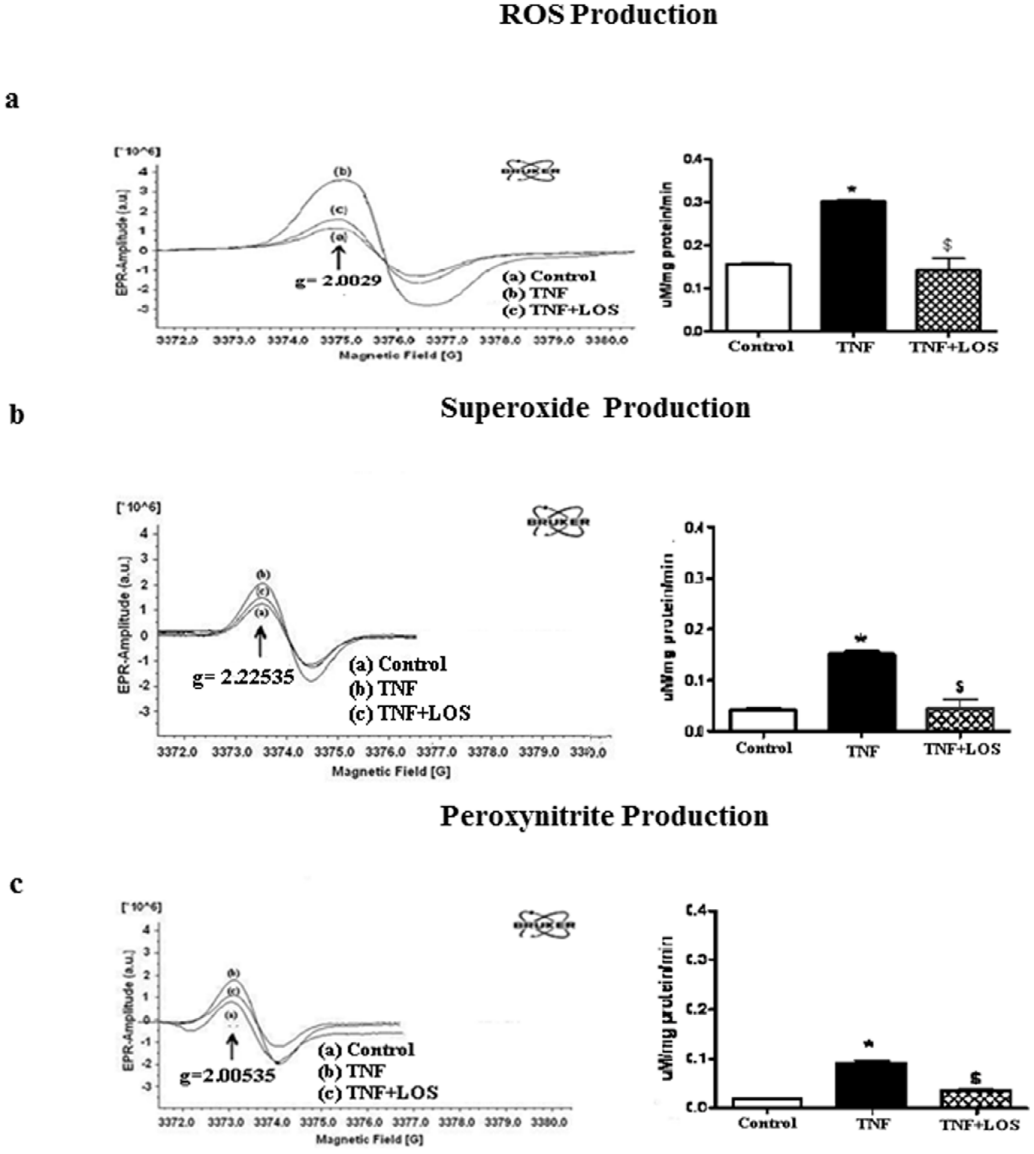
EPR spectra and their graphic interpretations are given. TNF administration significantly increased free radical production in LV tissue. Cytosolic a) total ROS, b) superoxide, and c) peroxynitrite production rates in rat cardiac tissues from each experimental group as measured by electron paramagnetic resonance spectroscopy. Administration of TNF to rats significantly increased production of all reactive species measured; LOS attenuated these increases. These results suggest that in the presence of an AT-1R antagonist, TNF cannot exert some of its detrimental effects.* p<0.05 vs. control; $ p<0.05 vs.TNF.

Total ROS, O_2_^•−^ and OONO^−^ production rates in LV tissue, as determined by EPR, were all significantly higher in LV tissues of TNF treated rats than in control and TNF+LOS groups (Figure 1a–1c ). Increases in ROS generation induced by TNF or ANGII were significantly inhibited by LOS. These results further support the ability of LOS to reduce oxidative stress in LV tissue.

### Gene and Protein Expression

**Figure 2:**
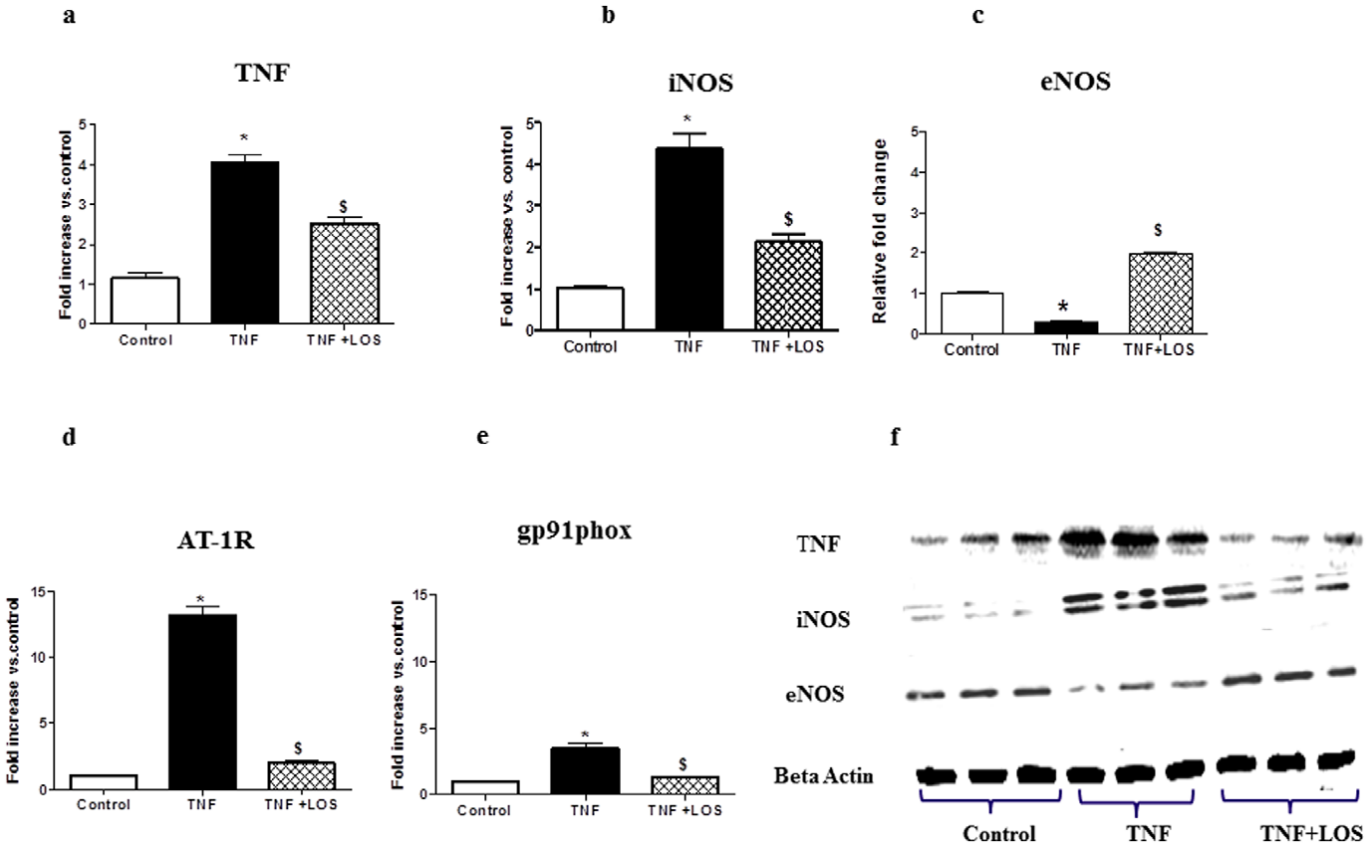
Effects of losartan treatment on mRNA expression of gene expression levels for a) TNF, b) iNOS, c) eNOS, d) AT-1R, and e) gp91phox as determined by real-time RT-PCR and protein expression levels of TNF, iNOS, and eNOS were measured by western blotting (Fig. 2f). Administration of TNF to rats induced significant increases in expression of proinflammatory and oxidative stress genes. Losartan attenuated these increases, thereby suggesting that TNF interacts with ANGII to cause some of its effects. * p<0.05 vs. control; $ p<0.05 vs.TNF.

Gene expression levels of TNF, iNOS, eNOS, AT1R and gp91phox were measured in the LV of rats by RT-PCR and protein expression levels of TNF, iNOS, and eNOS were measured by western blotting. TNF treatment resulted in significant increases in TNF and iNOS and a decrease in eNOS mRNA expression vs. controls, which was significantly attenuated with LOS treatment (Fig.2a–2c). AT-1R mRNA expression in LV was significantly increased in TNF-treated rats; LOS-treated rats demonstrated significant reductions in AT-1R expression compared to rats given TNF (Fig. 2d). These data suggest that ANGII plays an important role in the positive feedback involved in the upregulation of AT-1R in rats given TNF. TNF administration induced an increase in the mRNA levels of gp91phox (Fig. 2e) in the LV; this increase was prevented by LOS. Protein expression levels of TNF, iNOS and eNOS followed similar trends (Fig. 2f).

### Ultrastructure of Mitochondria

**Figure 3:**
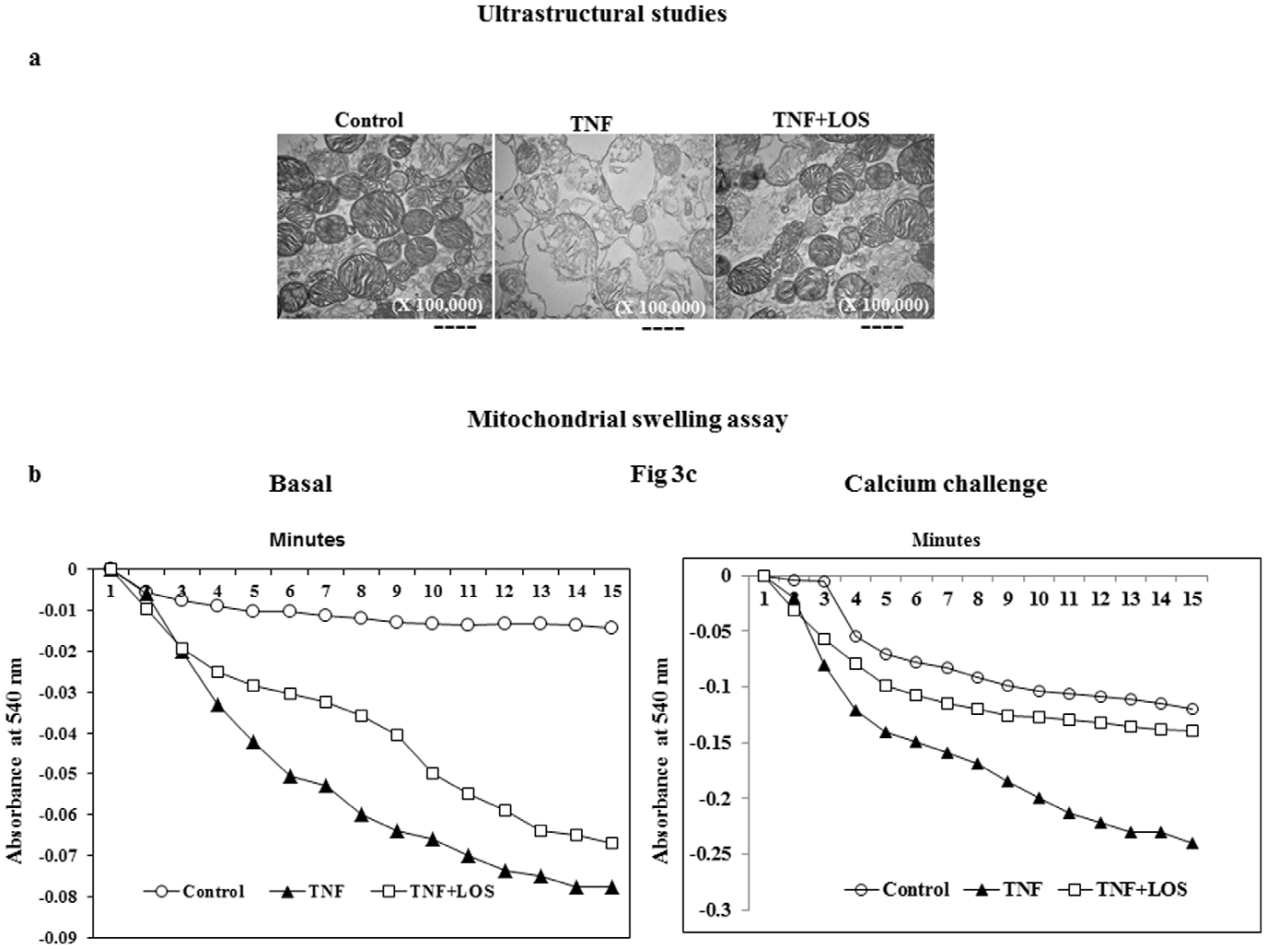
Representative electron micrographs of isolated rat heart mitochondria from each experimental group. a) Heart mitochondria from the TNF group demonstrated loss of outer and inner membrane structure, disordered cristae, and vacuolization. Note that the heart mitochondria from losartan-treated rats had a normal appearance. b) In the absence of and c) in the presence of a 50 mM calcium challenge. Heart mitochondria from rats given TNF demonstrated more swelling, which was indicative of disordered permeability transition.

Electron microscopic analysis of isolated LV mitochondria from the TNF group demonstrated swelled and disrupted mitochondria with loss of outer and inner membrane structure, disordered cristae, and vacuolization (Figure 3a). In contrast, mitochondria from the TNF + LOS treatment group had a normal appearance and showed maintenance of structural integrity.

### Mitochondrial Swelling

To determine the effects of LOS in rats given TNF, MPTP opening was induced against a 50 µM calcium challenge. When mitochondria are exposed to 50 µM calcium concentrations, especially when accompanied by oxidative stress, they undergo massive swelling. As demonstrated in Figures 3b and 3c, the extent of the swelling was higher in 50 µM Ca^++^ loaded mitochondria obtained from rats in the TNF group. The MPTP opened at a faster rate in the presence of Ca^++^ in rats given TNF than in the LOS-treated group, thus reinforcing the role played by the membrane permeability transition pore.

### Mitochondrial Superoxide and Hydrogen Peroxide Production

**Figure 4:**
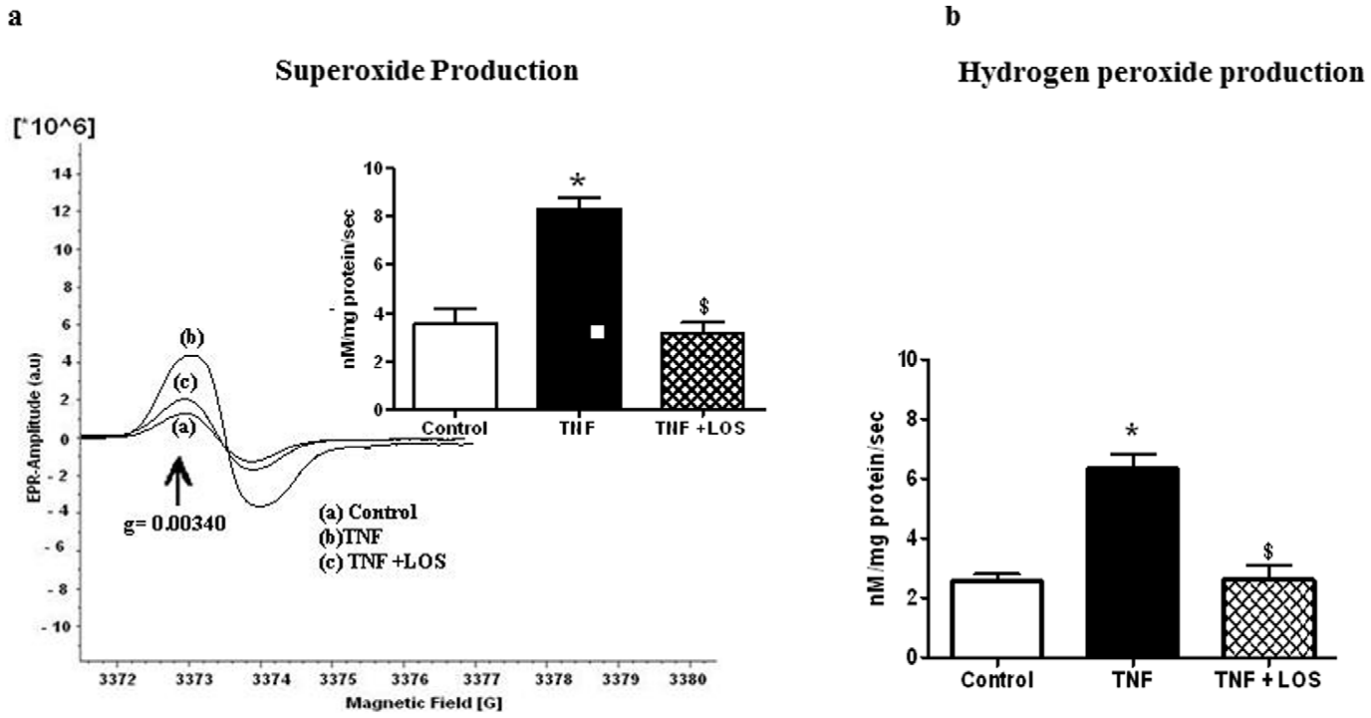
Effect of TNF on superoxide production rates and hydrogen peroxide production rates in isolated heart mitochondria from each experimental group as measured by EPR spectroscopy (a&b). Administration of TNF to rats resulted in significant increases in superoxide and hydrogen peroxide production rates. Losartan attenuated these changes. * p<0.05 vs. control; $ p<0.05 vs.TNF.

Mitochondrial O_2_^•−^ and H_2_O_2_ production rates were measured in rat heart mitochondria from each group. Mitochondrial O_2_^•−^ production (Figure 4a) and mitochondrial H_2_O_2_ production (Figure 4b) were significantly increased in rats given TNF; these increases were attenuated with concurrent LOS administration. These results support a role for ANGII in TNF-induced mitochondrial dysfunction.

### Mitochondrial Biogenesis

**Figure 5:**
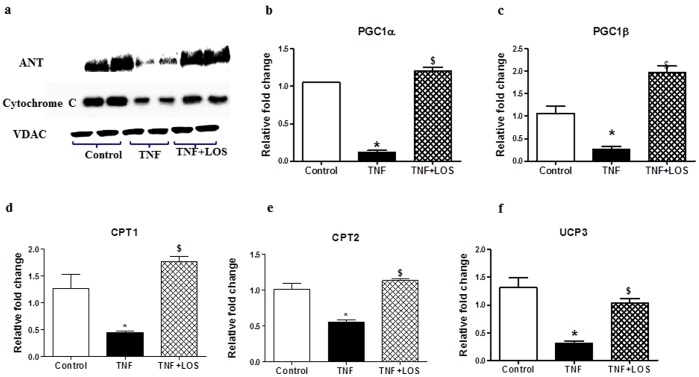
Representative western blot analysis of a left ventricle showing changes in expression of MPTP proteins from isolated mitochondria. TNF treated rats exhibited a significant decrease in adenine nucleotide translocator (ANT) and cytochrome C content when compared with the control and TNF+LOS groups. In the TNF+LOS treated group, ANT and cytochrome C protein levels were normalized (Fig 5a). Mitochondrial gene expression levels for b) PGC1α, c) PGC1β, d) CPT1β, e) CPT2, and f) UCP3 were all significantly decreased in animals given TNF; concomitant treatment with LOS attenuated these changes. * p<0.05 vs. control; $ p<0.05 vs.TNF.

We measured the expression of mitochondrial genes and proteins, including: ANT, cytochrome c, and VDAC, to further confirm that TNF and ANG II-impaired cardiac mitochondrial damage is mediated by TNF-induced oxidative stress. Expression of MPTP proteins in isolated mitochondria from TNF-treated rats, as determined by western blot, showed significant decreases in ANT and cytochrome C content compared with the control and TNF+LOS groups. In the TNF+LOS treated group, ANT and cytochrome C protein levels were restored to near that of controls (Figure 5a). Further, AT-1R blockade substantially increased MPTP proteins, and mRNA expression of PGC α and PGC β (coactivators of nuclear transcription factors, including PPARγ, PPARα, and PGC 2, Figures 5b &c), mitochondrial carnitine-palmitoyl transferase (CPT) Iβ and II (Figures 5d&e), and UCP 3 (Figure 5f) in TNF +LOS treated rats compared with TNF treated rats. These results further suggest that the interaction of TNF and ANG II induces cardiac mitochondrial oxidative damage, leading to depletion of mitochondrial genes, which in turn impairs mitochondrial respiratory complexes; consequently contributing to cardiac damage in TNF treated rats.

### Mitochondrial Complex Studies

**Figure 6:**
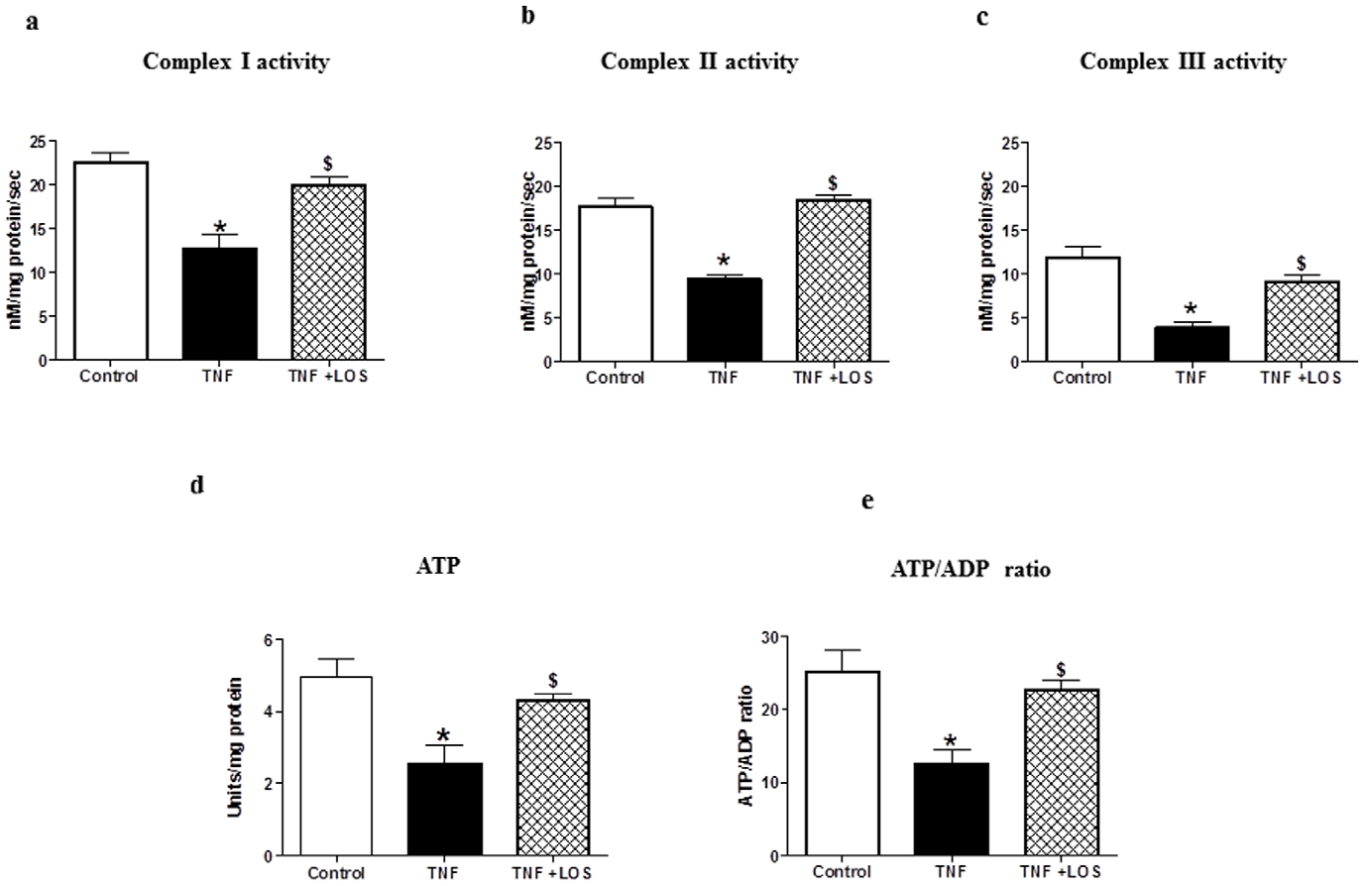
Effect of TNF on expression of mitochondrial respiratory complexes I, II and III measured by EPR spectroscopy. The activities of mitochondrial respiratory a) complex I, b) complex II, and c) complex III were assessed using EPR spectroscopy. TNF decreased the activities of all three complexes in isolated heart mitochondria; these results indicate a derangement in electron transport chain activity. Losartan attenuated these changes. * p<0.05 vs. control; $ p<0.05 vs. TNF d) ATP production rates and e) ATP/ADP ratios of heart mitochondria from each experimental group. TNF administration resulted in significant decreases in both ATP production rate and ATP/ADP ratio, which is suggestive of electron transport chain dysfunction. Losartan attenuated the changes seen in ATP production and ATP/ADP ratio. * p<0.05 vs. control; $ p<0.05 vs.TNF.

In order to further assess mitochondrial function, we measured the activities of respiratory complexes I, II, and III with electron paramagnetic resonance spectroscopy. Activities of these complexes decreased with TNF administration (Figures 6a–6c), thereby confirming that TNF can cause mitochondrial dysfunction. Interestingly, LOS treatment restored mitochondrial complex activity levels to near control levels, again suggesting a role for ANGII in the mitochondrial dysfunction that is caused by TNF.

### ATP Production and ATP/ADP Ratio

Since the main source of cellular energy, ATP, is produced in mitochondria, we examined the effects of TNF administration on ATP production and ATP/ADP ratio in rat hearts. In rats given TNF, ATP production rates were lower, thus, ATP/ADP ratio was lower; in LOS-treated rats, ATP production rates and ATP/ADP ratios were normalized (Figures 6d &e). These results indicate that TNF negatively affects the mitochondrial electron transport chain and that treatment with the AT-1R antagonist, LOS, can prevent some of these TNF-induced effects. These data further support the assertion that TNF interacts with ANGII to cause mitochondrial damage in this animal model.

## Discussion

**Figure 7:**
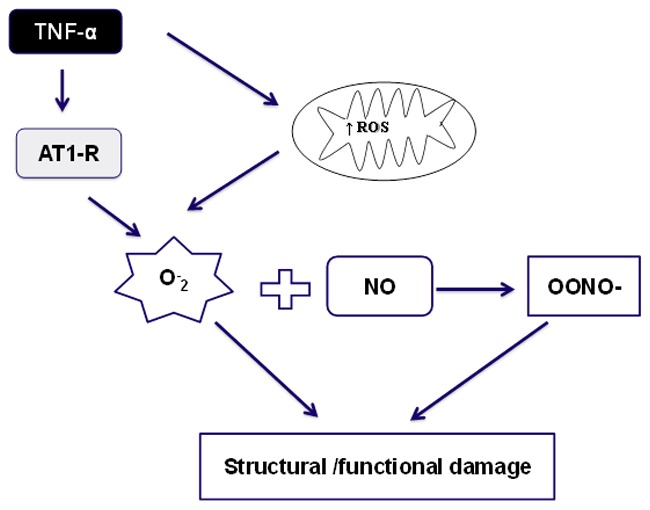
Schematic diagram showing TNF-induced mitochondrial dysfunction.

In our study, TNF treatment resulted in significant damage to the mitochondrial membrane and was accompanied by increased mitochondrial O_2_^•−^ production, decreased in ATP production (less capable of maintaining ATP levels during contraction in the heart mitochondria, (Figure7). This assertion is supported by several observations. Increased superoxide production, induced by TNF interacting with ANGII, contributes to mitochondrial dysfunction by depleting mitochondrial genes and proteins and decreasing respiratory complex activity, thereby influencing ATP synthesis and ultimately resulting in cardiac dysfunction. Administration of the ANGII type 1 receptor (AT-1R) antagonist losartan (LOS), to TNF-treated animals attenuates TNF-induced oxidative stress by modulating free radical production and increasing mitochondrial gene expression, which leads to a normalization of both mitochondrial complex activity and ATP synthesis, and thereby prevents cardiac dysfunction.

Our echocardiographic findings suggest that TNF decreases FS% and increases Tei index, both of which are indicative of diastolic dysfunction. We also observed increases in left ventricular diastolic (LVD) and systolic (LVS) dimensions, which indicate decreased left ventricular contractile function. Treatment with LOS improved left ventricular contractile function in our study; this could be due to reductions in cytokines and oxidative stress and possible increases in mitochondrial biogenesis gene expression and respiratory chain function. This is supported by our observation that LOS treatment decreased free radical generation both in the cytosol and mitochondria. Mitochondrial dysfunction may play an important pathogenic role in the progression of cardiac dysfunction. In the present study, we report markedly reduced LV tissue levels of specific mitochondrial enzyme activities for respiratory complexes I, II and III, enzymes critical to the generation of ATP. Furthermore, the reduced myocardial complex I,II and complex III levels correlated with the increased LV tissue TNF-α protein levels, suggesting an association between increased TNF-α and mitochondrial dysfunction. In the diabetic rat heart, mitochondrial dysfunction (decreased ATP synthesis rate and decreased State 3 respiration) was found to accompany diastolic dysfunction [26]. Further, TNF blockade in dogs with heart failure leads to a restoration of mitochondrial respiratory function in the left ventricle [27]. These observations, paired with our current observations, support a role for decreased mitochondrial bioenergetic function in contributing to diastolic dysfunction.

The roles of TNF and ANGII as potent inducers of oxidative stress in a number of cell types, including cardiac myocytes, are well established [28], [29]. There is also increasing evidence that inflammatory mediators are capable of upregulating various RAS components in a variety of mammalian tissues, including the heart [30]. It is well known that ANGII-generated ROS are produced by NAD(P)H oxidases [31], [32]. The NAD(P)H oxidases are regulated by a variety of pathophysiological stimuli, including ANGII, hemodynamic forces, and inflammatory cytokines [33], [34]. TNF is involved in myocardial release of free radicals via a self-amplifying process, as production of free radicals has been shown to further increase TNF [35]. Further, TNF interactions with ANGII seem to be mediated by an overproduction of ROS through modulation of vascular NAD(P)H oxidase activity and expression [36], [37].

Here, we report that AT-1R activation plays an important role in peroxynitrite production, and that AT-1R blockade reduces peroxynitrite production. Although LOS produces its beneficial effects via inhibition of AT-1R, the exact molecular mechanism contributing to these effects is still unclear. We have shown that TNF markedly decreased both endothelial nitric oxide synthase (eNOS) expression and mitochondrial biogenesis in TNF-treated rats. Thus, our findings are in agreement with those of earlier publications demonstrating that ANGII regulates NOS expression [36], modulates nitric oxide (NO) levels [38], and activates eNOS through AT-1R [39]. Consistent with previous studies, TNF increased AT-1R gene expression in rats given TNF, which led to upregulation of the NAD(P)H oxidase subunit gp91phox. This, in turn, stimulated ROS formation. These effects were attenuated by AT-1R blockers. Our results demonstrate that TNF interacts with ANGII via an increased functional AT-1R level in the LV, suggesting that, in addition to its direct cellular effects, ANGII might also enhance responsiveness to TNF and thus alter cardiac function.

TNF administration also upregulated the expression of inducible NOS (iNOS). This is probably because the iNOS gene is regulated by the transcription factor, NF-κB, which is known to respond to as well as induce, TNF production [40], [41]. Upon activation by TNF and/or ANGII, NF-κB translocate from the cytosolic compartment to the nucleus, where it binds to the iNOS gene promoter and ultimately triggers iNOS gene transcription. This process is mediated by kinase-mediated protein phosphorylation and requires disassociation between NF-κB and its native inhibitor IκB [42]. It is already known that induction of iNOS decreases NO bioavailability and increases superoxide and peroxynitrite/nitrotyrosine (NT) formation in the heart, which partially explains NO dysregulation in rats given TNF. Here, we report that TNF and ANGII interact to cause oxidative stress and result in the increased production of superoxide, which reacts with nitric oxide to produce peroxynitrite. This peroxynitrite can, in turn, cause mitochondrial damage by several mechanisms that include superoxide- and hydrogen peroxide-induced damage to respiratory complexes [43] and depletion of ATP synthesis.

Increased iNOS expression and peroxynitrite production formation (peroxynitrite, a potent oxidant and nitrating intermediate, is a product of the reaction of superoxide and NO derived from iNOS) have been observed in rats after TNF treatment. Higher NO production via iNOS has been shown to be associated with the pathogenesis of cardiac dysfunction and heart failure. We did detect an increase in iNOS mRNA and peroxynitrite formation in the hearts of rats treated with TNF, and these increases in iNOS expression, ROS production, superoxide generation, and peroxynitrite formation were accompanied by a marked loss of mitochondrial genes, whereas these changes were attenuated in TNF +losartan treatment with preservation of cardiac function. In the present study, we have also shown that TNF- α markedly decreased both eNOS expression and mitochondrial biogenesis in LV of TNF treated rats. Downregulation of eNOS seems to be the major molecular mechanism by which TNF-α affects mitochondrial biogenesis in these models; its effects on mitochondria can be reversed by treatment with losartan. These results suggest that TNF-α plays a relevant role in decreasing eNOS expression and mitochondrial biogenesis in metabolically active LV tissues of TNF treated animals.

Mitochondrial permeability transition pores (MPTP) are multiprotein complexes consisting of the adenine nucleotide translocase (ANT), voltage dependent anion channel (VDAC), cytochrome C, cyclophilin D, and several other ancillary proteins. The exact components that constitute the MPTP remain a subject of debate, although it is generally accepted that ANT and cytochrome C are major pore components [44], [45]. Increased oxidative stress can inhibit cytochrome c by increasing mitochondrial ROS production [46]. The redox state of the respiratory complexes is a major determinant of mitochondrial ROS production and is highest when the mitochondrial complexes (I, II and III) are highly reduced. In the present study, we demonstrate that chronic TNF treatment induces MPTP pore opening and causes mitochondrial swelling due to osmotic water entry, expansion of the inner membrane and consequent rupture of the outer membrane. As a consequence, MPTP proteins, such as ANT and cytochrome c, are released, and there is depletion of the activities of electron transport complexes I, II, and III and ANT due to the outer-membrane rupture. Treatment with losartan reduced mitochondrial damage and restored mitochondrial complex activity and prevented cardiac dysfunction.

Cardiac function is an energy driven process. In this study, we measured mitochondrial genes in the LV in TNF treated rats using real time RT-PCR. PGC1α is a coactivator of nuclear transcription factors, including PPARγ, PPARα, and nuclear respiratory factor 1 (NRF-1) and these genes are known to enhance mitochondrial activity. PGC-1α is abundantly expressed in the heart, and is known to 1) activate most genes of mitochondrial function and biogenesis, and 2) stimulate both fatty acid oxidation and oxidative respiration in cardiac tissue [47]–[49].In the present study, we have demonstrated that decreased expression of the PGC-1 gene caused significant deficiencies in cardiac energy reserves and function. Moreover, mitochondrial protein (ANT and cytochrome C; and respiratory protein Complex I, Complex II and Complex III ) levels, PGC-1α (which is regulated by eNOS) PGC -1β, CPT1, CPT 2 and UCP 3 were decreased in parallel, as were ATP production, thereby playing an important role in mitochondrial biogenesis. Thus, it is believed that abnormalities in mitochondrial biogenesis, mitochondrial number, and mitochondrial function contribute to altered energy metabolism, leading to cardiac dysfunction. Changes in mitochondrial morphology were also observed in tissues from TNF-treated rats. This study suggests that, in the presence of an inflammatory condition, mitochondrial biogenesis and mitochondrial fatty acid oxidation are negatively altered and contribute to altered energy metabolism, leading to cardiac dysfunction in the rat. Our data demonstrates that LOS treatment attenuates oxidative stress and can increase mitochondrial function directly, through up-regulation of electron transport chain activities, or indirectly, through a decrease in free radical generation, thereby restoring cardiac function. Taken together, these results demonstrate the presence of functionally significant interactions between RAS and TNF in the heart and suggest an important role for these interactions in the development of cardiac disease in this model.
